# Carbon Ion and Proton Therapy in Sacral Chordoma: A Systematic Review

**DOI:** 10.3390/jcm14175947

**Published:** 2025-08-22

**Authors:** Andrea Santoro, Riccardo Totti, Alessandro El Motassime, Cesare Meschini, Doriana Di Costa, Elena Gabrielli, Giulio Maccauro, Raffaele Vitiello

**Affiliations:** 1 Department of Orthopedics and Geriatric Sciences, Catholic University of the Sacred Heart, Largo Francesco Vito, 8, 00168 Rome, Italy; andrea.santoro01@icatt.it (A.S.); riccardo.totti01@icatt.it (R.T.); alessandro.elmotassime01@icatt.it (A.E.M.); doriana.dicosta01@icatt.it (D.D.C.); elena.gabrielli01@icatt.it (E.G.); giulio.maccauro@policlinicogemelli.it (G.M.); raffaele.vitiello@policlinicogemelli.it (R.V.); 2 Department of Orthopedics, Ageing and Rheumatological Sciences, Fondazione Policlinico Universitario A. Gemelli IRCCS (Scientific Istitute for Research, Hospitalization and Healthcare), Largo Agostino Gemelli 8, 00168 Rome, Italy

**Keywords:** hadron therapy, sacrum, chordoma, proton therapy, carbon ion therapy

## Abstract

**Background**: chordomas are characterized as locally aggressive yet infrequently metastasizing malignant neoplasms of bone, primarily arising in the axial skeleton, with a notable prevalence in the sacral region. En bloc resection is recognized as the standard treatment for sacral chordoma; however, its feasibility is not universally guaranteed. Therefore, definitive proton, carbon ion, or photon therapy is often utilized as an alternative to surgical intervention or as a (neo-)adjuvant measure in conjunction with surgery, owing to their role in enhancing local control. **Methods**: a search of PubMed yielded 127 articles, with 18 that were ultimately included in the review. This review aims to systematically evaluate clinical outcomes and complications associated with hadron therapy in cases of sacral chordomas. The review followed the PRISMA (Preferred Reporting Items for Systematic Reviews and Meta-Analyses) guidelines, including publication dates up to January 2025. **Results**: data extraction showed promising outcomes for patients treated with hadron therapy alone or when hadron therapy was used as an adjuvant for surgery, even if complications are described. The 5-year overall survival estimated from evaluating 10 of 18 articles was 82.4%, although some articles reported different results in shorter follow-up periods. Skin ulceration and pain were described in 323 (29%) and 186 (16%) patients, respectively. Chronic complications reported were sacral fractures, metastasis, rectal disorders, urinary disorders, and peripheral motor and sensory neuropathy. **Conclusions**: hadron therapy represents a highly effective and promising treatment for sacral chordomas. In cases of inoperable tumors, it has demonstrated outcomes comparable to surgery while significantly reducing treatment-related morbidity. Hadron therapy is also viable as adjuvant therapy and provides superior outcomes for patients who undergo surgery with positive margins compared to those treated with surgery alone, improving local control and overall prognosis.

## 1. Introduction

Chordomas are characterized as locally aggressive, albeit infrequently metastasizing, malignant neoplasms of bone, predominantly arising in the axial skeleton, with a notable prevalence identified in the sacral region [1]. The most prevalent sites of occurrence include the sacrococcygeal region (40–50%), the clivus (35–40%), and the vertebral bodies (15–20%) [2,3]. Chordomas represent rare, slowly growing, locally aggressive malignant bone tumors derived from remnants of the embryonic notochord. They were first described by Virchow in 1857, while Müller first postulated the hypothesis of their notochordal origin in 1858 [4,5,6]. These tumors account for 1–4% of all primary bone tumors, with an annual incidence estimated at 0.08 per 100,000 individuals. Chordomas exhibit a higher incidence among males, Caucasians, and individuals over the age of 40 years [7]. The clinical course of chordomas is notably aggressive, associated with a poor long-term prognosis, primarily due to increased local recurrence rates rather than metastatic potential. Reported local recurrence rates vary between 43% and 85%, with an overall survival rate of 61% at five years and 41% at ten years [3]. The presenting symptoms are highly variable and contingent upon tumor location. The localization at the skull’s base can cause compression of cranial nerves and brainstem structures, headaches, dysphagia, visual disturbances, and other neurological deficits. Chordomas of the spinal cord generally cause back pain, radiculopathy, motor weakness, sensory loss, and at least paralysis due to spinal cord compression. Sacral chordomas are characterized by symptoms like lower back pain, bowel and bladder dysfunctions and sexual impairments, weakness, and sensory abnormalities [8].

Chordomas are not generally regarded as responsive to chemotherapy, akin to numerous low-grade malignancies. Consequently, instances of chemotherapy response have been documented in patients exhibiting high-grade dedifferentiated chordomas, which account for less than 5% of all chordoma cases [9].

In the differential diagnosis of sacral masses, distinguishing chordomas from histologically similar lesions—such as chondrosarcomas, schwannomas, metastatic carcinomas, and other soft tissue tumors—can be challenging due to overlapping radiological and morphological features. A defining characteristic of chordomas is the overexpression of brachyury, a transcription factor encoded by the TBXT gene. While brachyury is essential for notochord development during embryogenesis, its expression ceases in normal adult tissues. In chordoma cells, however, brachyury remains aberrantly expressed, where it plays a critical role in promoting tumor cell proliferation, survival, and epithelial-to-mesenchymal transition (EMT)—mechanisms linked to tumor invasiveness and therapeutic resistance. Immunohistochemical and molecular analyses have consistently shown that brachyury is expressed across all histological variants of chordoma, including classical, chondroid, and dedifferentiated forms. In contrast, this protein is not detected in over 300 other benign or malignant tumor types, such as chondrosarcomas, reinforcing its utility as a highly specific diagnostic biomarker [10]. Functional experiments, particularly those utilizing RNA interference in the U-CH1 chordoma cell line, have demonstrated that inhibition of brachyury expression leads to significant reductions in cell proliferation, as well as morphological alterations and induction of cellular senescence. These results confirm that brachyury plays a critical role in maintaining chordoma cell viability [11].

Clinically, these findings carry substantial significance. The selective expression of brachyury in chordoma enhances diagnostic accuracy, especially when differentiating it from tumors with similar histologic appearances, such as chondrosarcoma or metastatic carcinoma. Beyond its diagnostic relevance, brachyury is now being evaluated as a therapeutic target. Various strategies are under development, including peptide-based vaccines designed to trigger immune responses against brachyury-positive cells, as well as small molecules and gene-silencing approaches aimed at reducing its activity. Because brachyury is largely absent in normal adult tissues, targeting it offers a tumor-selective approach with limited risk of off-target toxicity [11,12]. Among the molecular targets under investigation for chordoma, the Epidermal Growth Factor Receptor (EGFR) has also shown considerable promise. EGFR, a receptor tyrosine kinase involved in cellular proliferation, survival, and differentiation, is frequently found to be overexpressed or phosphorylated in chordoma samples, especially those from the sacral and skull base regions [13,14]. Although the activating mutations typically seen in other cancers, such as non-small cell lung cancer, are uncommon in chordoma [15], EGFR’s elevated expression and activation suggest it contributes to tumor development and progression. Preclinical research has confirmed that EGFR signaling supports the growth and survival of chordoma cells, and that inhibition of this pathway can impair tumor growth both in vitro and in vivo models [16]. Based on this, several early-phase clinical studies have evaluated EGFR-targeted therapies, including tyrosine kinase inhibitors (e.g., erlotinib, gefitinib, and afatinib) and monoclonal antibodies (e.g., cetuximab). Though the data remain preliminary, some patients—particularly those with elevated EGFR expression—have shown partial responses or disease stabilization with these agents [17,18]. Additionally, there is growing interest in combining EGFR inhibitors with other molecular therapies or radiation to improve outcomes and combat resistance mechanisms [19]. Ongoing clinical trials are also assessing new-generation EGFR inhibitors and pan-ErbB blockers to expand and refine treatment options for this challenging malignancy [20].

En bloc resection, introduced by Stener and Guntetberg in 1976, is recognized as the standard treatment for sacral chordoma; however, this approach is not applicable to other tumor locations, such as chordomas involving the skull base [21]. Along with symptoms consequent to tumor growth positioning, surgical treatment can involve delicate structures around the tumoral site. The sacral resection and kind of approach (anterior or posterior) often oblige surgeons to involve close structures (bowel, bladder, and nerve roots) with consequent symptoms: motor deficit (especially S1 resection), incontinence, and colostomy requirement [22]. The chordoma radiosensitivity allows the use of radiation as a valid therapy; however, proximity to close organs exposes patients to several risks and damages: the rectum, bladder, nerve roots, and sacrum are often involved. The linear tissue absorption profile of X-rays causes adjacent organ damage, and the angular change in radiation sources can not be expedient enough to avoid radiation damage [23].

In this scenario hadron therapy plays a fundamental role; it is an advanced form of oncological radiotherapy that uses charged particles—especially carbon ions and protons—instead of X-rays, commonly used in traditional radiotherapy. Peculiarity of these particles is their ability to release the majority of the energy at a specific depth within the tissues. After this peak, the quantity of energy released drops enormously. This phenomenon is called the Bragg peak, firstly described by Bragg in 1904, which facilitates precise tumor targeting while concurrently minimizing radiation exposure to surrounding healthy tissues [24,25,26].

Consequently, definitive proton or carbon ion radiotherapy is often employed as an alternative to surgical intervention or linear radiation therapy, moreover as a (neo-)adjuvant measure in conjunction with surgery, owing to their efficacy in enhancing local control.

While some physical differences exist between proton and carbon ion beams, particularly in relation to the widths of the penumbra and fragmentation tail, these particle beams are generally regarded as exhibiting comparable physical characteristics [27].

In this manuscript we present a review aimed to systematically evaluate clinical outcomes and complications associated with hadron therapy in cases of sacral chordomas.

## 2. Materials and Methods

The review followed the PRISMA (Preferred Reporting Items for Systematic Reviews and Meta-Analyses) guidelines [28], ensuring a thorough and systematic approach to data collection and analysis. This systematic review has also been registered with the International Prospective Register of Systematic Reviews (PROSPERO) under registration number 1002765.

### 2.1. Search Strategy

The search was performed across several online databases, including PubMed, Scopus, and Web of Science. The search string used in PubMed was as follows: (chordoma AND (sacral OR sacrum)) AND ((proton therapy) OR (protontherapy) OR (carbon therapy) OR (carbon ion) OR (carbon-ion) OR (hadron therapy) OR (hadron beam therapy) OR (particle therapy)).

We carefully examined the titles and abstracts of all retrieved articles to assess their eligibility for inclusion in the review. The criteria for inclusion were as follows: the studies must involve human adults, be published in English, and have publication dates up to January 2025. We included randomized trials, uncontrolled comparative trials, and case series.

When there was uncertainty, the full article was retrieved for further examination. The senior author and the content area experts then obtained the full text of all articles and reviewed them to minimize any bias that could arise from preconceived opinions about the studies and their findings. This process was further enhanced by following up on the reference lists of relevant studies to identify additional articles.

Two authors (A.E.M. and C.M.) independently reviewed the abstracts, obtaining the full texts for any abstracts that were inconclusive. Any differences between the reviewers were discussed, and if disagreements remained, the senior author (R.V. or G.M.) was consulted. The reference lists of the selected articles were manually checked to identify additional relevant studies. All the selected studies were then analyzed retrospectively by three authors (A.S., R.T., and D.D.C.), who extracted and entered the data into an Excel worksheet. Finally, the data sheet was reviewed by four authors (R.T., A.S., A.E.M., and E.G.), who reached an agreement on the extracted data. Additionally, the references of the identified papers were searched to find further relevant articles, and all journals were considered.

### 2.2. Inclusion and Exclusion Criteria

The eligibility criteria for our analysis were established to select studies that met high methodological and reporting standards. We included studies involving adult men and women aged 16 years or older who underwent hadrontherapy for sacral chordoma. Acceptable study designs comprised retrospective and prospective case series, controlled clinical trials, and both quasi-randomized and randomized controlled trials. Only studies with a minimum sample size of 10 patients; published in English; and reporting clinical, radiological, or complication outcomes with a follow-up period of at least 12 months were considered. Additionally, all articles had to be accessible through institutional or public journal databases.

To maintain the quality and relevance of our analysis, we excluded certain studies. This included research focused on pediatric populations (those younger than 16 years). Furthermore, articles that were not accessible through institutional resources or the British Library, studies with insufficient follow-up duration (less than six months), and those presenting duplicate data from previously published research were omitted. Lastly, studies that did not specify key outcomes, such as survival or recurrence rate, were excluded as well (Table 1).

Three reviewers (A.S., R.T., and D.D.C.) independently conducted the review process, evaluating the full texts of the selected articles to assess their eligibility and gather relevant data. When there was uncertainty regarding whether to include a study, the senior author made the final call. Additionally, three authors (E.G., G.M., and A.E.M.) independently evaluated the risk of bias using standardized criteria. Any disagreements were settled through discussion, and a supervising author (R.V.) was consulted when required.

### 2.3. Data Extraction and Analysis

**Figure 1:**
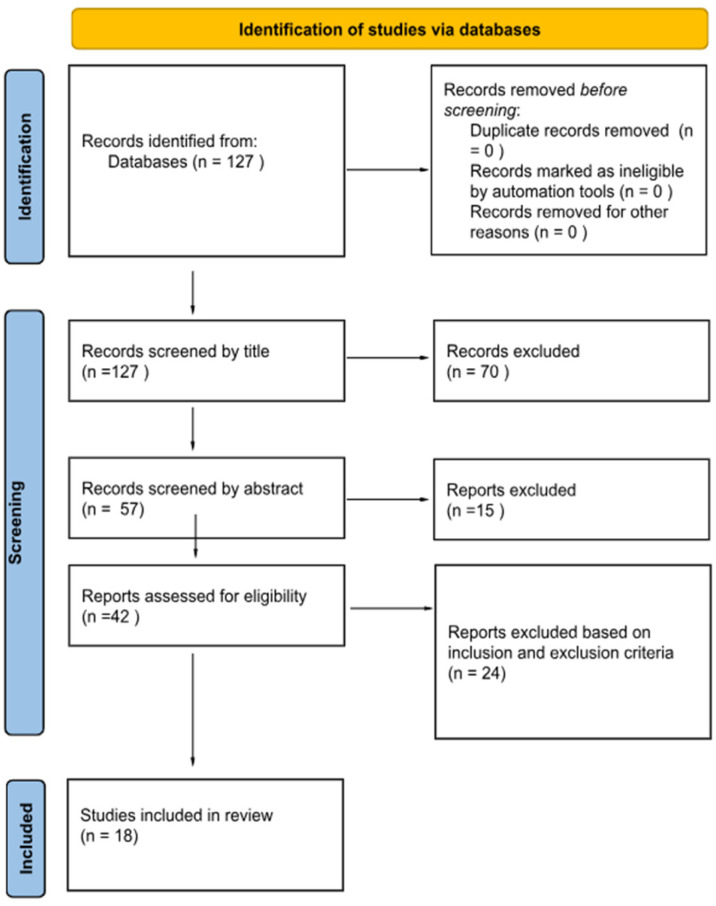
PRISMA flowchart.

The titles and abstracts were independently screened by two reviewers, C.M. and A.E.M. For abstracts that either met the inclusion criteria or caused uncertainty, full-text articles were obtained. These full texts were subsequently re-evaluated by the same two independent reviewers. Any discrepancies were resolved through assessment by the senior author, R.V. (Figure 1).

The methodological quality of each study was assessed using the Methodological Index for Non-Randomized Studies (MINORS) score, which offers a maximum of 24 points for comparative studies and 16 points for non-comparative studies [29]. Two authors, D.D.C. and E.G., independently assigned MINORS scores and reached a consensus on the final score.

Statistical significance was determined at a threshold of *p* < 0.05. The gathered data were analyzed and organized using the SPSS software version 30.0.0 (SPSS, Inc., Chicago, IL, USA). Categorical variables are displayed as frequencies and percentages, while continuous variables are presented as means with their standard deviations. All numerical data have been rounded to one decimal place for enhanced precision.

## 3. Results

The study analyses various parameters acquired from patients belonging to 18 articles in the period 2004–2025. Main parameters analyzed are number of patients, gender, use of surgical treatment, kind of Hadron therapy, radiation dose (Gy), adjuvant chemotherapy, follow-up period, survival, recurrences, time to recurrence, outcomes, and complications.

### 3.1. Demographics Data

Total number of patients examined in 18 articles consists of 1102 patients. All data reported in these 18 articles have been classified in several tables and processed using a “weighted average” to obtain results balancing the correct “weight” of each article.

Mean age calculated using the above-mentioned process was 64.6 years. The patient sample evaluated consisted of 739 (67%) males and 363 (32.9%) females (Table 2).

### 3.2. Therapy

As specified in 14 articles, not all patients underwent surgical treatment. In particular, 204 (18.51%) patients underwent surgical resection treatment; 140 (12.70%) of these had wide margin excision, and 64 (5.81%) had intralesional excision. As reported in these articles, 819 (74.31%) patients had no surgical treatment before hadron therapy.

All patients evaluated were administered proton therapy or carbon therapy. In 3 of 18 articles examined, proton and carbon therapy have been associated with other kinds of therapies, in particular: photon radiation therapy and hyperthermia [38,41,42]. Nevertheless, the insufficient sample of patients did not allow us to use the data belonging to these articles. The average dose used during hadron therapy was 66.7 Gy (E). No adjuvant chemotherapy has been administered to the patients examined in these 18 articles (Table 3).

### 3.3. Outcomes

Mean follow-up time was 51.91 months. Heterogeneous data reported about the overall survival period produced difficulties in estimation of the comprehensive “overall survival”. The 5-year overall survival estimated from evaluating 10 of 18 articles was 82.4%. Some articles reported different results in shorter follow-up periods: 100% of survival after 18 months [42]; 92.7% 3-year overall survival [44]; and 81% 4-year overall survival [32].

As highlighted in 10 studies, recurrences were described in 194 patients after hadron therapy treatment; the average time of recurrence was 29.6 months (±8.68 DS). (Table 3)

### 3.4. Complications

Several complications were highlighted after hadron therapy treatment. In particular, acute complications such as skin ulceration and pain were described in 323 (29%) and 186 (16%) patients, respectively. In contrast to skin ulceration, pain has been reported in acute and chronic development.

Chronic complications reported were sacral fractures, metastasis, rectal disorders, urinary disorders, and peripheral motor and sensory neuropathy.

Sacral fractures were described in 121 (11%) patients. Metastasis and peripheral motor/sensitive neuropathy were reported, respectively, in 97 (9%) and 171 (15.5%) patients.

Sacrum proximity to the gastrointestinal tract (particularly the rectum) and typical radiation susceptibility are the main causes of gastrointestinal disorders: rectal bleeding, constipation, and colostomy requirement. Rectal disorders have been reported in 58 (5.2%) patients.

As part of the gastrointestinal tract, the bladder is another target to avoid with proton/carbon beam; however, although peculiar features of hadron therapy, the bladder can be involved in radiation damage, causing cystitis, urinary retention, or incontinence. Urinary disorders have been reported in 71 (6.4%) patients (Table 4).

## 4. Discussion

Today, surgery is considered the choice treatment for sacral chordomas, in particular when complete resection with negative margins is feasible. However, surgery is often associated with significant morbidity due to the complex anatomical position and the proximity to highly delicate structures of the chordomas. The research on radical surgical removal at all costs is not always advisable. Sacral chordomas are also intrinsically resistant to conventional radiotherapy.

Hadron therapy shows significant advantages over conventional radiotherapy in the treatment of sacral chordomas. The peculiarity of hadrons is their physical property, especially the Bragg peak phenomenon. When positively charged particles pass through the tissues, they release most of their energy just before stopping: this phenomenon is called the Bragg peak. This allows for concentrating the radiation dose to the tumor and minimizing the exposure to healthy tissues.

This property is crucial for sacral chordomas because they are located near very delicate structures like the spinal cord and nerve roots. The occurrence of complications is significantly reduced in hadron therapy thanks to a reduction in the radiation exposure of surrounding tissues. The radiation is distributed over a larger area in traditional radiotherapy, photon-based, increasing the risk of collateral damage.

Since hadron therapy is a relatively recent introduction and remains largely inaccessible to many patients, there is limited data regarding the optimal timing for its use.

Yolcu et al. compared three patient cohorts: en bloc resection cohort, CIRT cohort, and National Cancer Database cohort (divided into 10 subcohorts). The results between en bloc resection and CIRT for sacral chordoma treatment revealed no statistically significant differences in overall survival (OS), progression-free survival (PFS), local tumor control, or distant metastases. However, peripheral motor neuropathy occurred more frequently in the en bloc resection group.

In the comparison with the national cohort, CIRT was associated with a higher OS rate than both primary radiotherapy and primary surgery with positive margins. No significant OS difference was observed between CIRT and surgery with negative margins. Patients who underwent adjuvant radiotherapy after a margin-positive resection had significantly lower OS compared to those treated with primary CIRT.

Uhl et al. conducted a study on 56 patients with sacral chordoma. A total of 41 received radiation therapy for a primary tumor and 15 for a recurrent tumor. Overall survival was 100% at a median follow-up of 25 months, but the 2-year local control (LC) rate was 85% for primary treatment and 47% for patients with recurrent tumors.

Nishida et al. analyzed 17 patients who underwent surgery or CIRT (10 surgery vs. 7 CIRT) and found a 5-year recurrence-free survival rate of 62.5% for the surgery group and 100% for the carbon ion RT group. Additionally, the 5-year disease-specific survival rate was 85.7% for the CIRT group compared to 53.3% for the surgery group.

Multiple studies have highlighted that male sex and primary tumors are associated with significantly better local control (Uhl et al., Seidensaall et al.).

Demizu et al. observed that younger age and a smaller planning target volume were significantly correlated with improved overall survival (OS).

Bostel et al. demonstrated that radiotherapy in cases of relapse was linked to lower local control rates compared to treatment in the primary setting. Regarding overall survival after radiotherapy, patient age and planning target volume size were identified as significant predictive factors. In contrast, other variables such as gross tumor volume, radiation dose, and treatment approach (carbon ion radiotherapy alone vs. in combination with photons) did not show a significant impact.

Aibe et al. found that cranial invasion of the tumor at or below the S2 level was a potentially significant prognostic factor for improved local progression-free survival (LPFS).

Imai et al. observed that patients with locally recurrent tumors following prior surgery had a significantly higher risk of developing distant metastases after CIRT compared to those who had not undergone any treatment before CIRT. When a tumor extends to S2 or above, achieving complete resection can lead to bladder and anorectal dysfunction as well as walking impairment. However, their findings indicate that tumor location and the irradiated area were associated with the onset of neurological complications. All the patients who experienced neurological issues had received CIRT at or above the S2 level.

Walser et al. analyzed 60 patients with histologically confirmed sacral chordoma who underwent either postoperative proton therapy (*n* = 50) or definitive proton therapy (*n* = 10). Tumor extension limited to the bone and a gross tumor volume exceeding 130 mL were identified as significant predictors of local recurrence. Multivariate analysis further confirmed that tumor confinement to the bone and complete tumor resection remained independent, favorable prognostic factors for reducing the risk of local recurrence.

From the data, favorable prognostic factors include younger age, male sex, treatment of primary tumors (both surgical and CIRT-based), a smaller planning target volume, and tumor extension limited to bone tissue. Tumor involvement at or above the S2 level is associated with higher rates of complications (motor neuropathy with walking difficulties, severe gastrointestinal and urinary issues), although these complications are less frequent in patients treated with CIRT compared to those undergoing surgery.

There is significant variability in treatment protocols.

Demizu et al. analyzed 219 patients who underwent CIRT for sacral chordoma, with 96% having no prior surgical resection. The range 100–500 mL (65%) was the most common planning target volume (PTV). The most frequently used dose-fractionation regimen was 67.2 Gy in 16 fractions (65%), while other approaches included 70.4 Gy in 16 fractions, 70.4 Gy in 32 fractions, and 79.2 Gy in 18 fractions.

Aibe et al. studied 33 patients with primary sacral chordoma treated with definitive proton beam therapy (PBT) at a dose of 70.4 Gy in 32 fractions. In cases where the gross tumor volume (GTV) was close to the intestine, a surgical spacer made of Gore-Tex sheets was used to create separation and minimize radiation exposure.

Imai et al. conducted a retrospective review of 38 patients with medically inoperable sacral chordomas treated with doses ranging from 52.8 to 73.6 Gray equivalents (median: 70.4) over 16 fixed fractions within 4 weeks at a single institution. Their findings indicated that tumors longer than 10 cm and a total dose exceeding 70 GyE were associated with a higher risk of severe neuropathy.

Serizawa et al. examined 34 patients with sacral chordoma who underwent CIRT with doses ranging from 52.8 to 73.6 Gy equivalents, administered once daily, four days per week (Tuesday to Friday), for a total of 16 fixed fractions over 4 weeks.

Further studies, particularly comparative studies, are needed to determine the optimal dose and fractionation schedule for treating non-surgical sacral chordoma.

Hadron therapy, like any treatment, is associated with complications.

Most commonly included mild complications are moderate pain; grade 1 or 2 skin reaction; and low-grade gastrointestinal symptoms like nausea, diarrhea, or cystitis. These side effects are generally manageable with supportive care and self-limiting.

Severe complications include peripheral motor neuropathy, which can result in muscle weakness, gait disturbances, and permanent functional deficits. Grade 3 or 4 skin toxicity, deep wound infections, and dehiscence can require surgical revision and can lead to severe inflammatory conditions, potentially resulting in patient exitus.

Sacral insufficiency fractures can develop months to years after the initial high-dose radiation treatment. Distant metastases and secondary tumors are relatively rare but still present [48]. Severe gastrointestinal or bladder dysfunction can require colostomy or urinary diversion.

## 5. Conclusions

In conclusion, hadron therapy represents a highly effective and promising treatment for sacral chordomas. In cases of inoperable tumors, it has demonstrated outcomes comparable to surgery while significantly reducing treatment-related morbidity. This suggests that hadron therapy could become a valid alternative for patients with unresectable tumors or those who decline surgical intervention. Hadron therapy is also viable as adjuvant therapy and provides superior outcomes for patients who undergo surgery with positive margins compared to those treated with surgery alone, improving local control and overall prognosis.

## Figures and Tables

**Table 1:** Inclusion and exclusion criteria.

Study Aspect	Inclusion Criteria	Exclusion Criteria
Types of studies	1. Retrospective and prospective case series 2. Controlled clinical trials 3. Quasi-randomized and randomized controlled trials 4. Non-blinded and blinded studies 5. Any European language	1. Articles not available through the British Library or our institutions’ online journal access
Types of participants	1. Adult men and women (age greater or equal to 16 years) 2. Minimum sample size of 10	1. Pediatric cases (age less than 16 years)
Types of interventions	1. Proton or Carbon ion therapy for sacral chordoma, associated or not associated with surgery	1. Articles regarding only surgical treatment 2. Articles regarding spine chordoma

**Table 2:** Overview of demographics.

Authors	Year	Type of Study	N° Patients	Age
Miladinovic et al. [30]	2025	Retrospective study	48	66
Yolcu et al. [31]	2022	Retrospective cohort study	47	58
Seidensaall et al. [32]	2024	Prospective, randomized phase II trial	82	63.5
Imai et al. [33]	2011	Retrospective study	95	66
Nishida et al. [34]	2011	Retrospective study	17	65
Imai et al. [35]	2010	Retrospective study	38	66
Imai et al. [36]	2004	Retrospective study	30	66
Serizawa et al. [37]	2009	Retrospective study	34	66
Park et al. [38]	2004	Retrospective study	27	56
Beddok et al. [39]	2020	Retrospective study	41	64
Demizu et al. [40]	2020	Retrospective study	219	67
Bostel et al. [41]	2020	Retrospective study	68	61
Tran et al. [42]	2019	Retrospective study	5	67
Bostel et al. [43]	2018	Retrospective study	56	61
Aibe et al. [44]	2017	Retrospective study	33	71
Imai et al. [45]	2015	Retrospective study	188	66
Evangelisti et al. [46]	2019	Retrospective study	18	64.7
Uhl et al. [47]	2015	Retrospective study	56	60

**Table 3:** Overview of outcomes.

Authors	Follow Up (Months)	Radiation Dose (Gy)	OS	PFS	Recurrence	Time to Recurrence (Months)
Miladinovic et al. [30]	49	-	-	79% (4 y)	-	-
Yolcu et al. [31]	68.1	-	>95% (5 y)	>95% (4 y)	9	-
Seidensaall et al. [32]	44.7	64	81% (4 y)	70% (4 y)	-	42.5
Imai et al. [33]	42	62	86% (5 y)	-	6	35
Nishida et al. [34]	49	-	-	53.3% (5 y)	-	-
Imai et al. [35]	80	62	86% (5 y)	54% (5 y)	4 local	13, 35, 48, and 59
Imai et al. [36]	30	70.4	52% (5 y)	-	-	13
Serizawa et al. [37]	At least 12	63	85.4% (5 y)	-	2	30.5
Park et al. [38]	91	71	82.5% (5 y)	60.5 (5 y)	Local control 71.7% 5 y, 57.5% 10 y	-
Beddok et al. [39]	46	71	80.3% (5 y)	-	8	46
Demizu et al. [40]	56	67.2	84% (5 y)	48% (5 y)	39 local recurrence, 59 regional/distant recurrence	-
Bostel et al. [41]	60	66	53% (5 y)	74% (5 y)	10% of local recurrence after 5 y. 9% distant metastasis	32.5
Tran et al. [42]	18	70	100% (1.5 y)	-	0	-
Bostel et al. [43]	35.5	66	53% (5 y)	74% (5 y)	-	-
Aibe et al. [44]	37	70.4	93% (3 y)	89.6% (3 y)	-	-
Imai et al. [45]	62	68	81% (5 y)	-	41	29 before 5 years
Evangelisti et al. [46]	23.3	70.4	100% (2 y)	-	2	18.5
Uhl et al. [47]	25	66	100% (2 y)	-	19	25

**Table 4:** Overview of complications.

Authors	Metastasis	Fracture	Skin Reaction	Urinary	Pain	Gastrointestinal	Peripheral Motor Neuropathy
Miladinovic et al. [30]	-	22 (45.8%)	-	-	-	-	-
Yolcu et al. [31]	14 (29.8%)	-	-	16 (34%)	-	9 (19.1%)	5 (10.6%)
Seidensaall et al. [32]	-	31 (37.8%)	78 (95.1%)	-	78 (95.1%)	-	77 (93.9%)
Imai et al. [33]	-	-	7 (7.4%)	-	-	-	15 (15.8%)
Nishida et al. [34]	-	-	1 (58.8%)	-	-	-	-
Imai et al. [35]	15 (39.5%)	1 (2.6%)	7 (18.4%)	5 (13.1%)	16 (42.1%)	4 (10.1%)	-
Imai et al. [36]	7 (23.3%)	-	6 (15.8%)	-	-	-	-
Serizawa et al. [37]	-	-	-	-	-	-	-
Park et al. [38]	7 (25.9%)	-	-	7 (25.9%)	6 (22.2%)	4 (14.8%)	-
Beddok et al. [39]	.	-	3 (7.3%)	0	-	6 (14.0%)	10 (25%) cauda equina
Demizu et al. [40]	-	-	6(2.7%)	-	3 (1.4%)	-	-
Bostel et al. [41]	-	33 (48.5%)	13 (19.1%)	23 (33.8%)	-	12 (18%)	-
Tran et al. [42]	-	1 (20%)	4 (80%)	-	5 (100%)	1 (20%)	-
Bostel et al. [43]	-	29 (51.8%)	-	-	10 (17.8%)	-	1 (1.8%)
Aibe et al. [44]	-	4 (12.1%)	15 (45.4%)	2 (6.1%)	21 (63.6%)	1 (3%)	-
Imai et al. [45]	54 (28.7%)	-	183 (97.3%)	-	-	4 (2.1%)	40 (21.3%)
Evangelisti et al. [46]	-	-	6 (33.3%)	1 (5.5%)	-	1 (5.5%)	8 (44.4%)
Uhl et al. [47]	-	-	-	18 (32.1%)	47 (83.9%)	17 (30.3%)	23 (41.1%)

## Data Availability

No new data were created in this study.

## Abbreviations

The following abbreviations are used in this manuscript:

CIRT	Carbon ion radiation therapy
OS	overall survival
PFS	progression-free survival
